# Predicting Transcatheter Heart Valve Expansion: A New Step Toward Durable Transcatheter Aortic Valve Implantation Outcomes

**DOI:** 10.1016/j.shj.2026.100853

**Published:** 2026-05-08

**Authors:** Antonin Trimaille

**Affiliations:** a Department of Cardiovascular Medicine, Nouvel Hôpital Civil, Strasbourg University Hospital, Strasbourg, France; b UR 3074, Translational Cardiovascular Medicine, CRBS, University of Strasbourg, Strasbourg, France

According to current guidelines, indications for transcatheter aortic valve implantation (TAVI) are expanding toward younger patients at lower surgical risk and with longer life expectancy.^1^^,^^2^ In this evolving landscape, a deeper understanding of valve-related outcomes and strategies aimed at improving long-term transcatheter heart valve (THV) durability has become more critical than ever. Among these determinants, the degree and symmetry of bioprosthesis expansion have emerged as key parameters closely linked to both clinical and hemodynamic outcomes after TAVI.

Asymmetric expansion and deformation of THVs are frequently observed following implantation, and they have been consistently associated with impaired valve hemodynamics and an increased risk of leaflet thrombosis regardless of whether balloon-expandable or self-expanding devices are used.^3^, ^4^, ^5^ In a landmark cardiac computed tomography (CT) study of 565 patients assessed 30 days after TAVI, more than half of implanted THVs were found to be significantly underexpanded.^6^ Importantly, nonuniform expansion, leading to frame deformation, asymmetric leaflet opening, and reduced neosinus volume, was strongly associated with the occurrence of leaflet thrombosis. In line with these observations, underexpansion of the Acurate valve has been linked to higher rates of the composite endpoint of death, stroke, and rehospitalization at 1 year.^7^ The deleterious consequences of THV underexpansion leading to leaflet thrombosis likely include leaflet pinwheeling, altered flow dynamics, unfavorable neosinus geometry, and complex interactions with the calcified native aortic valve.^8^, ^9^, ^10^ The growing body of evidence implicating thrombotic pathways in structural valve deterioration^10^ provides a mechanistic explanation for the observed association between THV underexpansion or asymmetric deformation with impaired valve durability.^3^^,^^11^

These observations have direct practical implications, as both underexpansion and asymmetric deformation may be mitigated through optimized device selection and procedural strategies, including meticulous preprocedural sizing, balloon predilation, postdilation, and bioprosthetic valve fracture in valve-in-valve procedures. In particular, a recent observational study found an association between balloon postdilation and a lower risk of Valve Academic Research Consortium-3 stage 2 or 3 hemodynamic valve deterioration and bioprosthetic valve failure at a median follow-up of 4 years.^12^ In this context, the ability to predict THV underexpansion during pre-TAVI planning would represent a major advance, enabling anticipation and individualized optimization of procedural strategy.

In this issue of *Structural Heart*, Akodad et al.^13^ report the results of a patient-specific computational model designed to simulate THV expansion during TAVI. The authors included 20 patients who underwent staged balloon postdilation for symptomatic THV dysfunction following either native-valve (n = 11) or valve-in-valve TAVI (n = 9), with comprehensive CT imaging available before TAVI, after TAVI, and following balloon valvuloplasty. Using finite element analysis, the investigators simulated both the initial valve deployment and subsequent postdilation. THV expansion was quantified as the ratio between the measured THV area (derived from CT or simulation) and the nominal manufacturer-defined area. The study demonstrated a significant, reproducible correlation between predicted and measured post-TAVI THV dimensions. A significant discrepancy between simulated and CT-derived measurements was observed only at the outflow level of balloon-expandable valves following the index TAVI procedure, whereas no such differences were seen after postdilation or in self-expanding devices. Notably, both predicted and measured degrees of THV expansion showed a strong inverse relationship with post-balloon aortic valvuloplasty mean transvalvular gradients. On this basis, the authors conclude that patient-specific computational modeling can reliably predict THV underexpansion during the index procedure and simulate the potential impact of postdilation.

This work addresses a highly relevant and timely question, as valve expansion is increasingly recognized as a central determinant of clinical outcomes, leaflet thrombosis, and long-term bioprosthetic durability.^8^^,^^10^ The strengths of the study include the sophisticated modeling approach and its validation against comprehensive, high-quality CT measurements. The authors should be commended for this important contribution, which provides a potentially new tool in the effort to address THV underexpansion.

Some limitations, however, warrant consideration. First, the small sample size (n = 20) and single-center design limit the robustness of the conclusions and preclude meaningful exploration of clinical endpoints associated with underexpansion, such as early hemodynamic valve deterioration^14^ or leaflet thrombosis.^6^ Second, the limited representation of self-expanding devices (n = 3), valve-in-valve procedures (n = 9), and complex anatomies (particularly the bicuspid aortic valve [n = 1]) restricts the generalizability of the findings to the broader TAVI population. Finally, the inclusion of only patients with THV dysfunction and underexpansion raises concern for selection bias. The absence of patients with adequate post-TAVI expansion makes it difficult to assess the specificity and negative predictive value of the model. In other words, while the model appears capable of predicting the degree of underexpansion, its accuracy in anticipating optimal expansion remains unproven.

Despite these limitations, the study has several important clinical implications. By demonstrating a robust correlation between predicted and observed THV dimensions, computational modeling could potentially be integrated into routine preprocedural planning. In cases of anticipated underexpansion, the Heart Team could proactively tailor the procedural strategy by optimizing valve sizing, considering predilation to modify native valve geometry, planning systematic postdilation to achieve symmetric deployment, or anticipating bioprosthetic valve fracture in the case of valve-in-valve procedures. Moreover, in patients presenting with clinically or hemodynamically significant underexpansion after TAVI, staged balloon postdilation remains a feasible, safe, and effective option to improve valve expansion and hemodynamic performance.^15^ In this setting, computational modeling may help identify patients most likely to benefit from such an intervention and facilitate balanced Heart Team discussions regarding risks and expected benefits. Finally, the observed relationship between THV expansion and postprocedural mean transprosthetic gradient represents one of the most compelling findings of the study. It underscores the direct hemodynamic consequences of suboptimal valve expansion and reinforces the need for meticulous procedural planning aimed at achieving the lowest possible residual gradient. As emerging evidence identifies elevated post-TAVI gradients as independent predictors of structural valve deterioration,^16^ achieving symmetric and complete valve expansion should be regarded as an important procedural objective rather than a secondary technical consideration.

Future multicenter studies applying this computational approach to larger and more diverse populations are needed to confirm its incremental value over real-time fluoroscopic operator assessment of THV expansion in the catheterization laboratory^11^ and determine whether incorporating predicted THV expansion into preprocedural planning ultimately translates into improved clinical outcomes and valve durability. Finally, beyond symmetry and expansion, operators should remain mindful that other procedural parameters influencing long-term THV performance exist, with commissural alignment, implant depth, and coaxiality also playing important roles in the quest for durable TAVI results.^10^^,^^17^

## Funding

The authors have no funding to report.

## Disclosure Statement

A. Trimaille has received a fellowship grant from Edwards Lifesciences.
